# New Insights into the Crossroads between EMT and Stemness in the Context of Cancer

**DOI:** 10.3390/jcm5030037

**Published:** 2016-03-12

**Authors:** Isabel Fabregat, Andrea Malfettone, Jitka Soukupova

**Affiliations:** 1 Bellvitge Biomedical Research Institute (IDIBELL), L’Hospitalet, Barcelona 08007, Spain; amalfettone@idibell.cat (A.M.); jsoukupova@idibell.cat (J.S.); 2 Department of Physiological Sciences II, University of Barcelona, Barcelona 08007, Spain

**Keywords:** EMT, MET, stem, Transforming Growth Factor-β (TGF-β), Snail, Zeb, Twist, Prrx, CD44, CD133

## Abstract

The epithelial-mesenchymal transition (EMT) is an example of cellular plasticity, where an epithelial cell acquires a mesenchymal-like phenotype that increases its migratory and invasive properties. Stemness is the ability of stem cells to proliferate in an asymmetric way that allows them to maintain the reservoir of undifferentiated cells with stem cell identity, but also to produce new differentiated cells. Initial works revealed that activation of the EMT program in epithelial cells induces the acquisition of stem cell properties, which in the context of cancer may contribute to the appearance of tumor initiating cells (TIC). However, a number of groups have recently reported that mesenchymal-epithelial transition (MET) is required for efficient metastatic colonization and that EMT may be not necessarily associated with stemness. In this review, we summarize recent findings that extend our knowledge about the crossroads between EMT and stemness and their relevance under physiological or pathological conditions.

## 1. Introduction

Cellular plasticity refers to the ability of cells to reversibly change their phenotype [1]. The epithelial-mesenchymal transition (EMT) is an example of that. EMT is a process where an epithelial cell acquires a mesenchymal-like phenotype that increases its migratory and invasive properties. This phenomenon takes place during both physiological and pathological conditions, particularly during embryogenesis and cancer [2]. The reverse process of EMT, called mesenchymal-epithelial transition (MET), occurs several times during embryogenesis [2], allowing cells to settle and differentiate into different organs and tissues. The hypothesis that mesenchymal migratory tumor cells would need to undergo MET to metastasize was proposed years ago [3]. However, the full understanding about how EMT and MET modulate metastasis continues being still matter of interest in different laboratories nowadays.

Stemness is the ability of stem cells (SC) to proliferate in an asymmetric way that allows them to serve as a reservoir of cells that maintain stem cell identity, but also as a source of new and more differentiated cells. Cancer stem cells have been proposed as the driving force of tumorigenesis and the seed of metastases [4]. Remarkably, activation of the EMT program in non-transformed epithelial cells confers properties of SC [5], which in the context of cancer would contribute to the appearance of tumor initiating cells (TIC) [6]. However, a number of groups have recently reported that MET is required for efficient metastatic colonization of mesenchymal-like migrating cells and that EMT might be not necessarily associated with stemness [7,8].

## 2. Epithelial-Mesenchymal Transition (EMT) and Stemness: General Overview

The EMT process is regulated by numerous signaling pathways that include the Transforming Growth Factor-β (TGF-β) family (includedins: BMP), fibroblast growth factor (FGF), Notch and Wnt, among others [9]. hepatocyte growth factor (HGF), interleukin-6 (IL-6) and other cytokines/chemokines derived from mesenchymal cells may promote de-differentiation, although their roles in triggering cancer cell EMT are not fully understood yet [9]. TGF-β is one of the strongest inducers of EMT under both physiological and pathological contexts [10]. It is considered a tumor suppressor factor in epithelial cells, inhibiting growth and inducing apoptosis. However, in advanced stages of tumorigenesis, cells acquire the capacity to overcome TGF-β-induced suppressor effects and respond to it undergoing EMT that facilitates migration and invasion [10]. Furthermore, TGF-β mediates production of mitogenic growth factors that stimulate tumor proliferation and survival [11]. TGF-β1 overexpression in human cancer correlates with tumor progression, metastasis, angiogenesis and poor prognostic outcome [12].

TGF-β and other EMT inducing factors activate different signals that finally converge in the expression of Transcription Factors (TFs) that regulate EMT (families of Snail, Zeb, and Twist, among others) [13]. EMT-TFs are tightly regulated by microRNA networks and epigenetic programs [13], long non-coding RNAs [14] or protein stabilization [15]. Loss- or gain-of-function experiments in cell and animal models revealed the involvement of EMT-TFs in both development and cancer [2,3,16]. Snail (*Snai1* gene), which was proposed as an essential regulator of EMT during embryonic development, is a strong repressor of transcription of the E-cadherin gene [17,18]. Epithelial cells that ectopically express *Snai1* adopt a fibroblastic-like phenotype and acquire invasive properties [17]. Snail protein is present in the invasive front of tumors, in which E-cadherin expression has been lost [17]. Its expression in human tumors inversely correlates with the grade of differentiation and is preferentially located in infiltrating carcinomas presenting lymph node metastases [19]. Specific silencing of *Snai1* in human carcinoma cells leads to a dramatic reduction of *in vivo* tumor incidence and growth rate and increases the sensitivity to chemotherapeutics [20]. In the same line of evidence, suppression of Twist (*Twist1* gene) in highly metastatic mammary carcinoma cells inhibits their ability to move from the mammary gland to the lung [21]. By the contrary, ectopic expression of *Twist1* results in loss of E-cadherin-mediated cell-cell adhesion and activation of mesenchymal markers, both events contributing to tumor metastasis [21]. In human breast cancers, high levels of Twist correlate with invasive lobular carcinoma, a highly infiltrating tumor type associated with loss of E-cadherin expression [21]. The later identification of Zeb1/2 and other basic helix-loop-helix (bHLH) transcription factors as inducers of EMT and potent repressors of E-cadherin in tumor progression [22] strongly suggested that the same molecules are used to trigger EMT during embryogenesis and tumorigenesis. The mechanisms underlying the expression of EMT-TFs in primary lesions remain elusive. Several phenomena associated with tumor progression (inflammation, metabolic stress, or abnormal activation of signaling pathways, such as those controlled by TGF-β, Wnt, and Notch, among others) are known to trigger expression of EMT-TFs [23]. Therefore, these pathways are particularly susceptible to gain-of-function mutations or constitutive signal activation that would force transition toward a mesenchymal phenotype [24]. Oncogenic events would also contribute to elicit these processes. Using oncogene-driven mouse mammary tumor models and cell-fate mapping strategies, Trimboli *et al.*, suggested that EMT in breast cancer would be favored by Myc-initiated events [25]. Whole-genome sequencing has revealed some oncogenic mutations in EMT-TFs [26], but they are not very frequent.

Remarkably, EMT-TFs display also oncogenic functions within the primary lesion that would affect tumor development. In this sense, EMT-TFs act as survival factors during development and tumorigenesis [27]. Slug protects hematopoietic progenitors from apoptosis after DNA damage [28]. Snail arrests cell cycle and confers resistance to pro-apoptotic signals, such as TGF-β, which correlates with higher levels of the anti-apoptotic Mcl-1 and Bcl-x(L) and lower expression of the pro-apoptotic Bim and Bmf [29,30]. A number of groups have recently reported an essential role for *Twist1* in tumor initiation that would be independent of its EMT-inducing activity, but related to its effects on inhibiting apoptosis [31].

Different groups initially revealed that the EMT process induced by TGF-β in epithelial cells correlates with the appearance of a less differentiated phenotype [32,33]. Mani *et al.*, later proposed that activation of the EMT program in non-transformed epithelial cells confers properties of SC [5]. In this sense, chronic treatment of human fetal hepatocytes with TGF-β induces a mesenchymal phenotype concomitant with loss in the expression of specific hepatic genes and appearance of SC markers, reminiscent of progenitor-like cells [34]. This process is reversible, since the mesenchymal stem-like cells re-differentiate to either hepatocytes or bile duct cells under controlled experimental conditions that provoke MET and re-expression of liver specific genes [34,35].

EMT may also confer stem-like properties on tumor cells. Morel *et al.*, showed that stem and tumorigenic characters of the cells were driven by EMT [36], using a mammary tumor progression model. Using a sensitive method to tag and track pancreatic epithelial cells in a mouse model of pancreatic cancer, Rhim *et al.*, found that disseminated tumor cells showed a mesenchymal phenotype and exhibited stem cell properties [37]. This would be consistent with the concept of a circulating (migratory) cancer stem cell (CSC) and supports the idea of a link between the EMT program and the stemness phenotype. Classical EMT-TFs, such as Snail/Slug, Twist or Zeb1/2, confer CSC properties [5,6,38]. TGF-β gives rise to tumor-initiating cells in the context of a cirrhotic liver [39]. Furthermore, TGF-β-induced EMT in liver tumor cells correlates with changes in the expression of stem markers [34,40,41]. However, the way in which EMT and stemness are connected, as well as its relevance for the metastatic process, are still controversial issues in tumorigenesis.

## 3. EMT and Stemness: Coupled, Antagonistic or Independent Processes?

First studies prompted to support the hypothesis that the same players that orchestrate EMT could be controlling stemness. Among others: (i) an elegant cooperative modulation of gene regulation by Snail and Slug mediates the acquisition of stem cell characteristics toward resisting radiotherapy- or chemotherapy-mediated cellular stress [42]; (ii) Bmi1, a polycomb protein that promotes self-renewal of certain stem-cell populations, is a direct transcriptional target of the EMT inducer, *Twist1* [43]; and (iii) Zeb1 links EMT activation and stemness maintenance by suppressing stemness-inhibiting microRNAs (miRNAs) [6]. Furthermore, different evidences reveal that the tumor suppressor p53 could regulate EMT-associated stem cell properties. Loss of p53 in mammary epithelial cells leads to lower expression of miR-200c that correlates with an increase in the expression of EMT and stemness genes [44]. p53 also inhibits the expression of the stem and progenitor-cell-associated protein Nestin, restricting cellular plasticity and tumorigenesis in liver cancer [45].

Different groups have recently revealed that stemness-related signaling pathways, such as Wnt, have also been involved in some aspects of the EMT program. The β-catenin/T-cell Factor 4 (TCF4) complex binds directly to the *Zeb1* promoter and activates its transcription [46]. The expression of stem-related genes could also be provoking the acquisition of an EMT phenotype. In particular, cluster of differentiation (CD) 44 or CD133 have been involved not only in the acquisition of stem properties, but also in the switch to a more mesenchymal, migratory phenotype [47,48]. The standard form of CD44 (CD44s) regulates the TGF-β-mediated mesenchymal phenotype in liver tumor cells [40,47]. Overexpression of CD44 is associated with low expression of E-cadherin, high expression of vimentin, a high percentage of phospho-Smad2 positive nuclei and poor prognosis in hepatocellular carcinoma (HCC) patients [47]. A self-enforcing feed-back loop that employs CD44s to activate *Zeb1* expression renders tumor cell stemness independent of external stimuli, since Zeb1 further promotes CD44s isoform synthesis [49].

However, embryonic stem cells (ESC) are epithelial-like and MET is required for the nuclear reprogramming of fibroblasts with the Yamanaka factors (Sox2, Klf4, Oct4 and Myc) [50]. Furthermore, different groups have indicated that EMT can suppress major attributes of human epithelial TIC [51]. Indeed, constitutive overexpression of the transcription factor Snail1 in epithelial/TIC-enriched populations addresses a mesenchymal gene program, but suppresses their self-renewal and metastatic phenotypes. Conversely, knockdown of EMT factors in mesenchymal-like cancer cell subpopulations induces a gain in epithelial features and properties of TICs [51]. Ocaña *et al.*, demonstrated that loss in the expression of the homeobox factor Prrx1 (an EMT inducer that confers migratory and invasive properties) is required for cancer cells to metastasize *in vivo*. Lower levels of Prrx1 allow cells to revert to a more epithelial phenotype, concomitantly with the acquisition of stem cell properties [7]. In the same line of evidence, Tsai *et al.*, proposed that activation of Twist1 is sufficient to promote carcinoma cells to undergo EMT and disseminate [8]. However, in distant sites, turning off Twist1 is essential to allow disseminated tumor cells to proliferate and form metastases [8]. Similarly, breast cancer metastases in an inducible *Snai1* expression mouse model are highly dependent on *Snai1* expression only when the expression is transient [52]. Forced, continuous expression of *Snai1* led to a decrease in lung metastasis [52].

**Figure 1:**
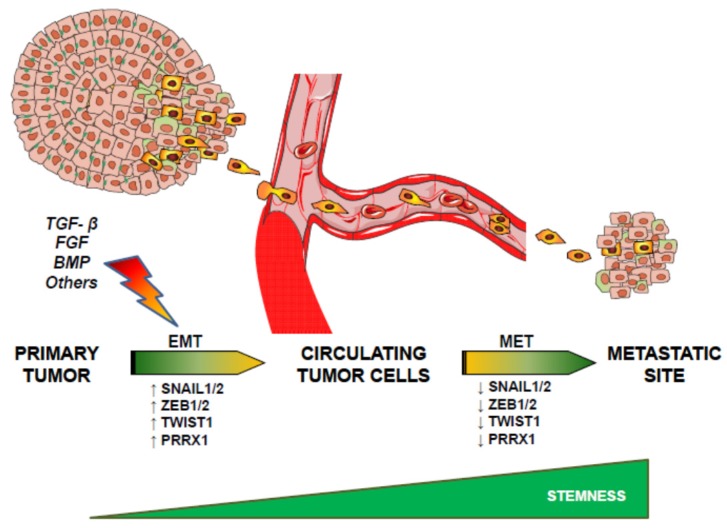
Sequential epithelial-mesenchymal transition (EMT) and mesenchymal-epithelial transition (MET) allows tumor cells to acquire the capacity to migrate and later colonize tissues for an efficient metastatic process. See text for details.

Another possibility could be that EMT and stemness are independent processes. As such, Slug and Sox9 define the stem cell state in normal mammary glands, but whereas Slug is mainly involved in the induction of EMT, Sox9 is responsible for the entry into the stem cell phenotype [53]. Authors suggest that the EMT program is important for inducing entrance into the stem state, but it is not sufficient on its own to induce this change in differentiated luminal cells. Instead, activation of an additional genetic program, in the present case through expression of Sox9, is required to work in concert with the EMT program to induce stemness [53]. Additional evidences have suggested that Slug is required for Sox9 stabilization and both cooperate to promote cancer SC and metastasis [54]. Remarkably, Schmidt *et al.*, have proposed that although a cross-talk between EMT and stemness exists, their actions are somehow antagonistic and attenuation of the EMT process is required for the full acquisition of stem cell properties [55]. Indeed, these authors suggested that Twist1 activation may prime cells for stem-cell-like properties, but these attributes only emerge and stably persist following Twist1 deactivation. In line with this idea, Stankic *et al.*, have demonstrated that during metastatic colonization, *Id1* expression induces a MET and stem-like phenotype specifically in breast cancer whose mesenchymal state is dependent on the Id1 target *Twist1*. In contrast, this does not occur at the primary site, where this state is controlled by *Snai1* [56]. Thus, Twist1-mediated EMT is a prerequisite for subsequent Id1-induced stemness during metastatic colonization. These results together would indicate that EMT contributes to the acquisition of stem cell properties, even though turning off key master regulators of EMT, such as Snail, Twist or Prrx1, is necessary to acquire TIC properties in the metastatic site (Figure 1).

Remarkably, the metastatic niche could be formed even before the arrival of the metastatic cells, since tumor-derived exosomes up taken by organ-specific cells would prepare the pre-metastatic niche [57,58]. Macrophage migration inhibitory factor (MIF) is highly expressed in pancreatic ductal adenocarcinomas (PDACs)-derived exosomes and primes the liver for metastasis [57]. Exosome proteomics reveals distinct integrins expression patterns [58]. The association to a specific organ is dependent on the integrins expression [58]. This suggests that exosomal integrins could be used to predict organ-specific metastasis.

## 4. Epithelial Plasticity: The EMT Transient State

**Figure 2:**
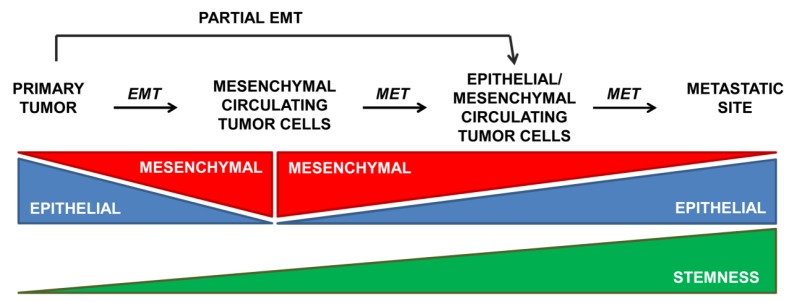
The activation of the EMT program may present different threshold levels that couple or uncouple EMT from stemness ability. See text for details.

During embryonic development or pathological situations, cells undergo a partial EMT that concurs with simultaneous expression of both epithelial and mesenchymal genes [1]. A partial EMT is also activated as a response to injury in the adult organism, such as during renal fibrosis, where this intermediate phenotype is defined as the final stage [59,60]. In cancer, the transient nature of EMT allows mesenchymal cancer cells to partially reverse to the epithelial phenotype to colonize new tissues and organs [7,8,51]. In view of these results, Brabletz [61] has recently postulated two different scenarios for EMT during metastasis: (i) EMT and stemness are linked processes that lead to the formation of migratory CSCs, such as those associated to Twist1 that induces both EMT and stemness properties (although Twist1 down-regulation appears to be required for recovery of some epithelial characteristics that are necessary for the metastatic process) [8]; and (ii) EMT and stemness are independent events, even antagonistically regulated, such as those associated with Prxx1 that confers EMT and migratory properties but suppresses stemness [7]. In this last model, down-regulation of Prrx1 is required for the acquisition of stem properties and metastatic colonization. Interestingly, Ombrato and Malanchi have recently suggested that only an early EMT program would correlate with CSC capability via a gain of epithelial plasticity, whereas an advanced EMT status may lead to a less flexible mesenchymal phenotype [62]. In this “EMT-gradient” model, the activation of the EMT program may present different threshold levels that couple or uncouple EMT from stemness ability (Figure 2). The initial activation of the EMT program would allow epithelial cell to reprogram its phenotype to acquire both migratory and stem-like features. However, the acquisition of a fully committed mesenchymal phenotype (such as that one addressed by Prrx1) may represent an alternative differentiation program in which stem-like features are lost. Microenvironmental signals might instruct these two possible phenotypes: more mesenchymal during invasion, more epithelial/stem during metastatic colonization [62]. The partial mesenchymal state found in different carcinomas is under the control of multiple EMT programs [13] that differ in EMT-TFs usage, epigenetic and metabolic reprogramming, as well as paracrine and autocrine signals [63]. Jolly *et al.* [64], by using a theoretical framework that couples the core EMT and stemness modules, presented OVOL (a transcription factor that regulates embryogenesis through its involvement in the differentiation of epidermal progenitor cells) as an example of a modulating factor that can fine-tune the EMT-stemness interplay.

It is worth mentioning that another consequence of this EMT gradient state could be the differential expression of stem markers by CSCs, depending on their epithelial-mesenchymal phenotype. During an intermediate EMT stage, both epithelial and mesenchymal stem genes can be expressed, as it occurs after treatment of epithelial HCC cells with TGF-β, where a mixed epithelial-mesenchymal phenotype is acquired [40]. HCC epithelial cells express high levels of EpCAM and CD133, but very low levels of CD44 and CD90. However, HCC cells that show a mesenchymal phenotype and autocrine over-activation of the TGF-β pathway do not express EpCAM or CD133, but they express CD44 and CD90. When epithelial cells respond to TGF-β, the transient EMT state offers the highest variability in terms of stem gene expression, since cells simultaneously express EpCAM, CD133, CD44 and CD90 [40] . Further work will be necessary to understand if this transient state that allows expression of both epithelial and mesenchymal stem genes confers relevant advantages in terms of functional stem properties. As suggested by Medema, stemness would be also a flexible—rather than fixed-quality of tumor cells that can be lost and gained [4].

## 5. EMT and Stemness in the Crossroads of Chemotherapy Resistance

**Figure 3:**
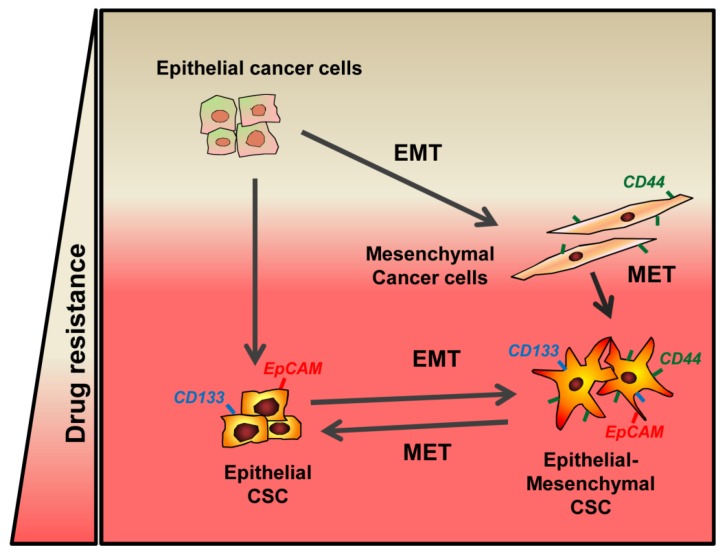
EMT and Stemness in the crossroads towards chemotherapy resistance. See text for details.

Finally, but not less importantly, we would like to mention that EMT and stemness, each separately or coordinately, confer resistance to chemotherapy in cancer cells. From first studies about the role of EMT in tumor cells, it was evidenced that Snail and Slug induce resistance to chemotherapeutic agents [27] by antagonizing p53-mediated apoptosis [42]. In the same line of evidence, later studies demonstrated that other factors, such as Notch and Twist, are involved in tumor resistance through its capacity to induce EMT [65,66,67]. EMT-inducing transcription factors, such as Twist, Snail and Slug, up-regulate ATP-binding cassette (ABC) transporter genes [68], although a recent study demonstrates that the expression of ABC transporters is dispensable for the induction of chemoresistance by Twist and Snail [69]. Regardless of the specific mechanisms, two recent different works in genetically engineered mice reveal that although the contribution of EMT process to metastasis might be less than previously anticipated, it is necessary for the induction of chemoresistance in breast and pancreatic cancer [70,71] (Figure 3).

CSC population is highly heterogeneous and undergoes dynamic clonal modification during the metastatic cascade, chemotherapeutic treatment, dormancy and relapse [72]. Therefore it is not surprising that CSCs contribute to chemoresistance in various tumors [73,74]. Since the EMT process is implicated not only in promoting cell motility and invasion, but also in the generation of the so-called cancer stem cell phenotype, both EMT and stemness may contribute to drug resistance. Correspondingly, up-regulation of Snail in breast cancer cells led to the acquisition of stem-like character concomitant with an increase in chemoresistance [75]. Expression of some stem genes, such as CD133 [76,77,78] or CD44 [79], confers chemoresistance. Variant CD44 isoforms induce resistance to therapeutic drugs, which is linked to the activation of the Src-family tyrosine kinase lyn [79] or Nanog/Stat3 signaling [80], among others [81]. Treatment of HCC epithelial cells with TGF-β does not only induce EMT, but also is able to up-regulate CD44 [47]. Under these circumstances, CD44 impairs the HCC cell response to sorafenib-induced apoptosis [40]. Similarly, cancer stem-like spheres induced from de-differentiated HCC-derived cell lines showed increased expression of stemness markers, such as CD44, and possessed resistance to anti-cancer drugs [82].

Different groups have reported that the miRNAs play important roles in drug-resistant mechanisms and are, therefore, considered as targets for cancer therapy [83,84]. In many cases, the miRNAs are controlling EMT and stemness. As an example, overexpression of miR-124 targets Prrx1 and radiosensitizes human colorectal cancer cells [85]. In contrast, down-regulation of miR-223 reverses EMT, decreases migration and invasion of pancreatic cancer cells and sensitizes them to gemcitabine [86]. The family of miR-200 influences the EMT phenotype by regulating the expression of Zeb1/2. Its re-expression is expected to induce not only the reversion of EMT, but also self-differentiation, inhibiting CSC self-renewal capability [84]. Interestingly, miR-200c is down-regulated in trastuzumab-resistant cells that have undergone EMT and show elevated TGF-β signaling. Restoration of the miR-200c expression in these cells suppresses TGF-β signaling and its target Zeb1, counteracting trastuzumab resistance [87]. An innovative approach was used in a study by Meidhof *et al.*, to identify new strategies to overcome chemoresistance in pancreatic cancer [88]. They found that downregulation of miR-203 induces Zeb1 activation; therefore, the restoration of miR-203 would induce sensitivity against chemotherapy. Additionally, mocetinostat (an epigenetic drug that inhibits histone deacetylase activity-HDAC) was identified to interfere with miR203 expression, restoring sensitivity to chemotherapy [88].

## 6. Concluding Remarks

The EMT phenotype is an example of cellular plasticity, where different intermediate situations may occur. This is the reason for so many different, sometimes controversial, findings about the role of this process in tumor progression. There is no doubt about the essential role of EMT in mediating cell migration and survival. However, when circulating TICs show a mesenchymal phenotype, re-acquisition of epithelial features is required for metastasis, a process that converges with gain of cell tumor initiating capacity. Response of tumor cells to chemotherapeutic agents is somehow dependent on their mesenchymal-TIC phenotype. Therefore, the analysis of the molecular mechanisms that regulate all these processes remains being an attractive focus of investigation in the search for new tools to fight tumor metastasis, which at the end is the major cause of death by cancer.

A clear challenge for the following years will be the development of new animal models for better understanding the function of EMT-TFs in stemness. Most of the previous studies involved ectopic expression of EMT-TFs, or their regulatory factors, often at non-physiological levels. Interestingly, the Weinberg group has recently developed genetically engineered knock-in reporter mouse lines, where they underscore profound differences in the transcription-regulating activities of the endogenously encoded Slug and Snail, proving that normal stem cells and TICs of the same tissue-of-origin could arise from different cellular compartments and exploit different molecular signaling pathways [89]. Indeed, the development of sophisticated fate-mapping experiments in animal models is required to completely understand the cross-talk between EMT/MET and stemness and its relevance in cancer progression and metastasis.
